# Antibacterial, antifungal and antioxidant activities of whole plant chemical constituents of *Rumex abyssinicus*

**DOI:** 10.1186/s12906-021-03325-y

**Published:** 2021-06-05

**Authors:** Irene Chinda Kengne, Léonel Donald Tsamo Feugap, Abdel Jélil Njouendou, Claudia Darille Jouogo Ngnokam, Mahamat Djamalladine Djamalladine, David Ngnokam, Laurence Voutquenne-Nazabadioko, Jean-De-Dieu Tamokou

**Affiliations:** 1 Department of Biochemistry, Research Unit of Microbiology and Antimicrobial Substances, Faculty of Science, University of Dschang, P.O. Box 67, Dschang, Cameroon; 2 Department of Chemistry, Research Unit of Applied and Environmental Chemistry, Faculty of Science, University of Dschang, P.O. Box 67, Dschang, Cameroon; 3 Department of Biomedical Science, Faculty of Health Sciences, University of Buea, P.O. Box 12, Buea, Cameroon; 4 Groupe Isolement et Structure, Institut de Chimie Moléculaire de Reims (ICMR), CNRS UMR 7312, Bat. 18 B.P. 1039, 51687 Reims Cedex 2, France

**Keywords:** *Rumex abyssinicus*, Antimicrobial, Antioxidant, Multiresistant strains, Membrane leakage, Dehydrogenase activity

## Abstract

**Background:**

Antibiotic resistance has contributed to the burden of infectious diseases both in the hospital and community setting, and represents a great threat to public health. Previous studies have revealed the role of reactive oxygen species as intermediate mediators of tissue damage, following antibiotherapies, indicating the need of associating antioxidants to these treatments. Therefore, the present work was designed to study the antibacterial, antifungal and antioxidant activities of extracts and compounds from *Rumex abyssinicus* Jacq. (Polygonaceae), as well as to investigate the antibacterial mechanisms of action of the most effective agents.

**Methods:**

The plant extracts were prepared by maceration in organic solvents followed by column chromatography of the EtOAc fraction and purification of different fractions which led to the isolation and characterization of pure compounds. The antimicrobial activities of the extracts/compounds and their combinations with ciprofloxacin and fluconazole were evaluated using the broth microdilution method by determining the minimum inhibitory concentration (MIC) and minimum microbicidal concentration (MMC). The effects of the extracts on the bacterial cell membrane and microbial respiratory chain dehydrogenase enzyme activity were determined by spectrophotometric methods. Antioxidant activity was evaluated using 1,1-diphenyl-2-picrylhydrazyl (DPPH) and gallic acid equivalent antioxidant capacity (GAEAC) assays.

**Results:**

Chrysophanol (**1**), physcion (**2**), Ergosta-6,22-diene-3,5,8-triol (**3**), emodin (**4**), 6-hydroxyemodin (citreorosein) (**5**), chrysophanein (**6**) and physcionin (**7**) were isolated from EtOAc fraction of *R. abyssinicus* and displayed different degrees of antimicrobial activities (MIC = 8–256 μg/mL). The MeOH extract and compounds **2** and **4** exhibited synergistic effects with ciprofloxacin and fluconazole. Compounds **1**, **2** and the combined mixture of **6 + 7** displayed the highest antioxidant activity (GAEAC = 83.38–106.03 μg/mL).

**Conclusion:**

*R. abyssinicus* is a potential source of antibacterial, antifungal and antioxidant agents. The antibacterial mechanisms of action of the MeOH extract and compound **2** are due to disruption of the cytoplasmic membrane and inhibition of the microbial respiratory chain dehydrogenase enzyme activity. To the best of our knowledge, this is the first report of test samples and ciprofloxacin / fluconazole association against MDR strains. The observed activity of the isolated compounds against bacteria and fungi including MDR strains deserves further exploration.

**Supplementary Information:**

The online version contains supplementary material available at 10.1186/s12906-021-03325-y.

## Background

The increasing appearance of resistant pathogenic bacteria and fungi to synthetic antimicrobial agents represents an alarming threat to public health. The most commonly encountered antibiotic-resistant bacteria, methicillin-resistant *S. aureus* (MRSA), vancomycin-resistant *Enterococci* (VRE), and penicillin and cephalosporin-resistant *Streptococci* (PCRS) have contributed to the burden of infectious diseases both in the hospital and community setting [1]. Majority of the classical antibiotics today sold in the market have major disadvantages resulting from the side effects on patients and the developed multiple drug resistances by the pathogenic microorganisms [2]. Hence, a growing interest in the discovery of new natural antimicrobial agents has been observed, with the objective to combat these resistant pathogens while avoiding or minimizing the undesirable consequences and side effects related to the consumption of synthetic antibiotics [3]. Previous studies have demonstrated detrimental side effects of bactericidal antibiotics such as quinolones, aminoglycosides while, β-lactams caused mitochondrial dysfunction and reactive oxygen species (ROS) overproduction in mammalian cells, leading to oxidative damage to DNA, proteins, and membrane lipids [4]. Therefore, associating antioxidant with antibiotic therapy seems to be a strategy to mitigate or prevent side effects.

Reactive oxygen species are oxygen-derived free radicals, metabolic products arising from endoplasmic reticulum and mitochondria of various cells. Free radicals which are delivered as a consequence of typical biochemical responses in the body are implicated in diabetes, atherosclerosis, ageing, cancer, inflammation, immunosuppression, neurodegenerative disorders and ischemic heart disease [5]. Free radicals are proven to be highly toxic to pathogens and they are used as a means to prevent tissue colonisation by the microorganisms. Thus, the production of free radicals is highly elevated during infection and this situation can cause oxidative stress; which further complicates the patient’s condition. Secondary metabolites of plants such as flavonoids and terpenoids play an important role in the defense against free radicals and pathogenic microorganisms [6]. Previous studies have shown that the use of plant-derived medicines have increased tremendous interest in the search of alternative antimicrobial and antioxidant agents because of the perception that they cause minimal adverse effects and have a long history of use in folk medicine for the treatment of infectious diseases and oxidative stress conditions [7, 8]. However, the combination of antioxidant and antimicrobial agents has gained wide acceptance within the pharmaceutical industries [9]. In fact, combining two or more compounds could be more effective for the improvement of antioxidant and antimicrobial activities and could offer a synergistic effect. The fact that flavonoids, terpenoids and saponins can improve the susceptibility of some bacteria to certain antibiotics have been demonstrated in many studies [10, 11]. Natural products of higher plants may possess a new source of antimicrobial and antioxidant agents with possibly novel mechanisms of action [12]. Hence, three levels of interactions are involved: interaction with the outer cellular components; interaction with the cytoplasmic membrane and interaction with cytoplasmic constituents. Natural products can act with the bacterial cells at one level or all three levels of interaction to produce their antimicrobial activities. Their systematic and methodical screening may result in the discovery of novel active principles to overcome resistance mechanisms in multidrug resistant microorganisms.

It is well documented that plants belonging to *Rumex* genus possess suitable medicinal properties, which are based mainly on the presence of anthraquinones, flavonoids and terpenoids [13]. *R. abyssinicus* Jacq. (Family: Polygonaceae) commonly known as Spinach Rhubarb, is a large herbaceous perennial plant that grows up to 4 m in height. This plant is mainly found in tropical Africa especially in the drier areas. *R. abyssinicus* is locally used as astringent, purgative, taeniafuge, depurative and hemostatic [14]. The plant is also used in the management of breast cancer, gonorrhea, liver diseases, hypertension and hemorrhoids [14]. The fresh or dried plant is applied externally to treat cough, pneumonia, wounds, rheumatism, sores and scabies [14]. An extract of rhizome is consumed to control mild forms of diabetes and, with water, to cure stomach-ache [14]. The crude extracts of *R. abyssinicus* have been shown to possess antibacterial [15, 16], anticancer [16], antiviral [15], anti-inflammatory [15, 17], antioxidant [18], wound healing [17], antimalarial [19], diuretic and analgesic [20] activities. Up to date, there has been no report on the antibacterial, antifungal and antioxidant activities of compounds isolated from *R. abyssinicus*, although there is an ample ethnobotanical claim for these properties. Therefore, the present work was designed to study the antibacterial, antifungal and antioxidant activities of extracts and compounds from *R. abyssinicus* as well as to investigate the mechanisms of antibacterial activity of the most effective agents. Interactions of the methanol extract/compounds from *R. abyssinicus* and antibiotics against bacterial and yeast species were also investigated.

## Methods

### General experimental procedures

#### NMR analysis

The ^1^H and ^13^C-NMR spectra were recorded on a Bruker Avance III 600 spectrometer equipped with a cryo-platform (^1^H at 600 MHz and ^13^C at 150 MHz). 2D NMR experiments were performed using standard Bruker microprograms (Xwin-NMR version 2.1 software). All chemical shifts (*δ*) are reported in parts per million (ppm) with the solvent signal as reference relative to TMS (*δ* = 0) as internal standard, while the coupling constants (*J*) are given in Hertz (Hz). Deuterated solvents, methanol (CD_3_OD), dimethyl sulfoxide (DMSO-*d*_6_), and chloroform (CDCl_3_) were used as solvents for the NMR experiments.

#### Chromatographic methods

Column chromatography was run on Merck silica gel (VWR, France) 60 (70–230 mesh) and gel permeation on Sephadex LH-20 (VWR, France), while TLC was carried out on silica gel GF254 pre-coated plates and the spots were visualized by an UV lamp multiband UV-254/365 nm (ModelUVGL-58 Upland CA 91786, U.S.A) followed by spraying with 50% H_2_SO_4_ and then heating at 100 °C.

### Sample collection

The whole plant of *Rumex abyssinicus* Jacq. was collected in February 2018 from the wild in Dschang, western region of Cameroon. The botanical identification was carried out by Victor Nana, a botanist of the National Herbarium of Cameroon, where a voucher specimen (N° 50,551/HNC) has been deposited.

For the collection of plants, no specific permits were required for the described field studies. For any locations/activities, no specific permissions were required. All locations of plant collection were not privately-owned or protected in any way and the field studies did not involve endangered or protected species.

### Extraction and fractionation

The whole plant material of *R. abyssinicus* was air-dried at room temperature and ground into fine powder. This dried powder (4.5 kg) was extracted at room temperature with methanol (3 × 20 L, 72 h) to yield 200 g of crude methanol extract after evaporation of solvent under reduced pressure. A part of this crude extract (195 g) underwent a differential solubilization with H_2_O/EtOAc (300 mL/500 mL) followed by H_2_O/*n*-BuOH (300 mL/500 mL). After evaporation of each solvent under reduced pressure, we obtained 50 g of EtOAc and 18 g of *n*-BuOH extracts respectively.

### Isolation of compounds

A part of the EtOAc fraction of *R. abyssinicus* (45 g) was subjected to silica gel column chromatography eluted with *n*-hexane-EtOAc (95:5 → 80:20) followed by EtOAc-MeOH (95:5 → 70:30) gradient graduated elution to yield seventy fractions of 400 mL each. These were combined on the basis of TLC profiles to yield eight major fractions A-H (A: 1–3; B: 4–10; C: 11–22; D: 23–28; E: 29–35; F: 36–44; G: 45–63; H: 64–70). Fraction A (4.0 g) underwent column chromatography on silica gel with the *n*-hexane-EtOAc system (95:5) to yield compounds **1** (15 mg) [21] and **2** (17 mg) [22]. Sephadex LH-20 gel column chromatography of fraction C (1.9 g) led to two sub-fractions (C_1_ and C_2_). Purification of sub-fraction C_1_ (500 mg) by silica gel column chromatography (*n*-hexane-EtOAc, 90:10 → 80:20) resulted in compound **3** (15 mg) [23]. The sub-fraction C_2_ (300 mg), was purified on Sephadex LH-20 gel column using MeOH as eluent to give compound **4** (40 mg) [22]. After Sephadex LH-20 gel column using MeOH, fraction D (3.74 g) led to three sub-fractions D_1_, D_2_ and D_3_. Purification of D_3_ (400 mg) sub-fraction by silica gel column chromatography with *n*-hexane-EtOAc (85:15) gave compound **5** (11 mg) [24]. Recrystallization of fraction G (5 g) afforded a mixture of two compounds **6** + **7** (10 mg) [25] which unfortunately, was not separated by silica gel column chromatography method.

### Antimicrobial assay

#### Microorganisms

Five bacteria and two yeasts were tested for their susceptibility to the studied samples. The studied microorganisms were three Gram-positive (*Staphylococcus aureus* ATCC25923, methicillin sensitive *S. aureus* MSSA01 and methicillin resistant *S. aureus* MRSA03) and two Gram-negative (*Pseudomonas aeruginosa* ATCC27853, *Shigella flexneri* SDINT) bacteria and two yeast strains of *Candida albicans* ATCC10231 and *Cryptococcus neoformans* H99. These microorganisms were taken from our laboratory collection. The bacterial and fungal species were maintained on agar slant at + 4 °C and on nutrient agar (NA, Conda, Madrid, Spain) and Sabouraud Dextrose Agar (SDA, Conda) slants respectively, prior to any antimicrobial test.

### Determination of minimum inhibitory concentration (MIC) and minimum microbicidal concentration (MMC)

MIC and MMC values were determined as described earlier [26]. The test samples were dissolved in dimethylsulfoxide (DMSO). The negative control well consisted of 195 μL of MHB or SDB and 5 μL of the standard inoculum. The MICs were visually assessed and were considered as the lowest sample concentration inhibiting the growth of the microorganism. The lowest concentrations that showed no visual growth after the sub-culturing were considered as the minimum microbial concentration (MMCs). Ciprofloxacin (Sigma-Aldrich, Steinheim, Germany) and fluconazole (Merck, Darmstadt, Germany) were used as positive controls for bacteria and yeasts, respectively. All tests were performed in triplicate.

### Combined effect of antibiotics and MeOH extract, compounds 2 or 4

The antimicrobial effects of a combination of samples (MeOH extract, compounds **2** and **4**), which exhibited the highest antimicrobial activities, and antibiotics (ciprofloxacin and fluconazole) were assessed by the checkerboard method as previously described [27]. The inoculum was initially prepared as described above. The test microorganisms were inoculated into a 96-well microtitre plates and a serial dilution of two antimicrobial agents: antibiotic and MeOH extract, compound **2** or **4**. Each well consisted of unique combination of test sample and antibiotic concentrations. The plates were then incubated for 24 h at 37 °C. The analyses were performed in triplicates. And the antimicrobial agents interactions were evaluated by calculating the fractional inhibitory concentration (FIC) indices. The FIC is defined as follows: MIC of antibiotic tested in combination/MIC of antibiotic tested alone + MIC of extract/compound tested in combination/MIC of extract/compound tested alone. The FIC index is interpreted as FIC ≤ 0.5: synergistic effect, 0.5 < FIC ≤1: additive effect, 1 < FIC ≤2: indifferent effect, and FIC > 2.0: antagonistic effect.

### Antibacterial mechanism studies

#### Cell membrane leakage assay

The alteration of cell membrane of *P. aeruginosa* and *S. flexneri* was evaluated by measuring the optical densities at 260 nm and 280 nm of the bacterial suspensions in the presence and absence of MeOH extract and compound **2** using the method described by Karsha and Lakshmi [28].

#### Evaluation of the sugar leakage through membrane of bacteria

10 mL of the bacterial suspension containing 10^8^ CFU/mL were inoculated into MeOH extract or compound **2** at ½ MIC, MIC and 2MIC and incubated at 37 °C under agitation at 150 rpm for 12 h. After incubation, the mixture was centrifuged at 12,000 rpm and the supernatant was collected. The concentration of reducing sugar was determined spectrophotometrically at 550 nm using 3–5 dinitro-salicylic acid (DNS) [29].

#### Assay of respiratory chain dehydrogenase enzyme activity in the bacteria

Cellular bioenergetic is a domain with promising future in the development of novel antimicrobials. Several studies have evaluated the bioenergetics of various bacterial pathogens, which explain the abilities of electron donor and acceptor utilisation, and the regulation of components of electron transport chain in bacteria. In this assay, the effect of the most effective agents on respiratory chain dehydrogenase enzyme activity of pathogenic bacteria as a test for mechanism of antibacterial action was performed. The dehydrogenase activity assay was performed using 2,3,5- triphenyl tetrazolium chloride (TTC) as previously described [30]. The TTC serves as the artificial electron acceptor and is reduced to red coloured triphenyl formazan (TPF). The assay was carried out with 3 ml of nutrient broth-glucose-TTC medium, supplemented with varying concentrations of MeOH extract or compound **2** in 20 mL screw-capped test tubes. The TPF produced after each exposure period (0, 30, 60 min) was extracted in 4 mL of amyl alcohol and determined spectrophotometrically at 500 nm. The amount of formazan produced was determined from a standard dose-response curve (R^2^ = 0.9983). Dehydrogenase activity was expressed as the amount of TPF formed (μg) per amount of dry cell weight of cell biomass (in mg). Data were expressed as the mean ± standard deviation.

### Antioxidant assay

#### Gallic acid equivalent antioxidant capacity (GEAC) assay

The GEAC test was done as previously described [31] with slight modifications. In a quartz cuvette, to 950 μL acetate buffer (pH =5.0, 100 mM), the following were added: 20 μL laccase (1 mM stock solution), 20 μL test sample, 10 μL ABTS (2,2′-azinobis(3-ethylbenzothiazoline-6-sulfonic acid) (74 mM stock solution). The purification of laccase from *Sclerotinia sclerotiorum* was done according to the protocol described [32]. The sample concentrations in the assay mixture were 800, 400, 200, 100, 10 μg/mL for the extracts and 200, 100, 50, 25, 12.5, 6.25, 3.125, 1.56 μg/mL for the isolated compounds. The content of the generated ABTS^**●+**^ radical was measured at 420 nm after 240 s reaction time and was converted to gallic acid equivalents by the use of a calibration curve (Pearson’s correlation coefficient: *r* = 0.997) constructed with 0, 4, 10, 14, 28, 56, 84 μM gallic acid standards rather than Trolox. Experiments were done in triplicate.

#### Diphenyl-1-picrylhydrazyl (DPPH) free radical scavenging assay

The free radical scavenging activity of extracts and compounds was evaluated according to described methods [33]. The EC_50_ (μg/ml), which is the amount of sample necessary to inhibit by 50% the absorbance of free radical DPPH was calculated [33]. Vitamin C was used as a standard control. All the analyses were carried out in triplicate.

### Cytotoxicity assay

Three male Wistar rats (*Rattus novergicus*), aged 10–12 weeks and weighing 230 to 240 g were used. These animals were bred in the animal house of the University of Dschang, Cameroon. Efforts were also made to minimize animal suffering and to reduce the number of animal used in the experiment. All the rats were anaesthesized via intraperitoneal injection of the mixture of ketamine (50 mg/ kg body weight, BW) and xylazine (10 mg /kg BW), in a dose that is commonly used for operation purposes. Subsequently the unconscious animals were decapitated swiftly and the whole blood (10 mL) was collected by cardiac puncture into a conical tube containing Ethylene Diamine Tetra Acetic Acid (EDTA) as an anticoagulant. Erythrocytes were obtained by centrifugation at room temperature for 10 min at 1000 x *g* and were washed three times in PBS buffer [34]. The cytotoxicity was evaluated as previously described [34].

### Statistical analysis

Data were analyzed by one-way analysis of variance followed by Waller-Duncan Post Hoc test. The experimental results were expressed as the mean ± Standard Deviation (SD). Differences between groups were considered significant when *p* < 0.05. All analyses were performed using the Statistical Package for Social Sciences (SPSS, version 12.0) software.

## Results

### Chemical composition

**Fig. 1:**
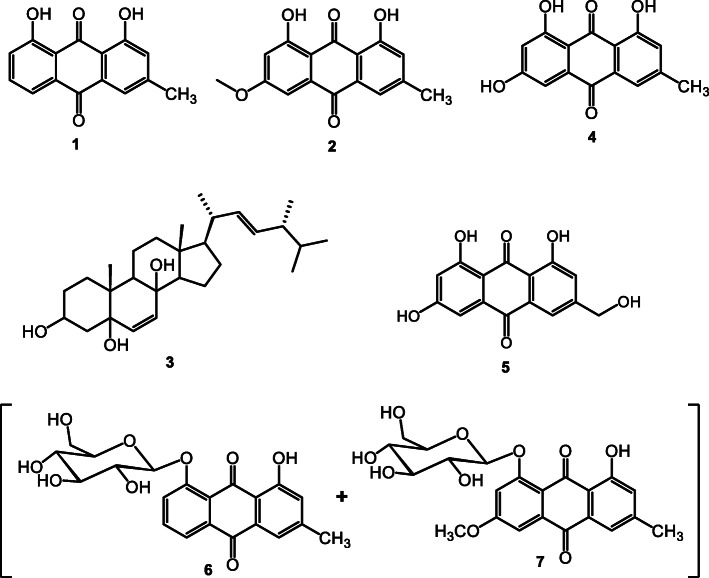
Chemical structures of compounds isolated from *R. abyssinicus* (1–7): 1: Chrysophanol; 2: Physcion; 3: Ergosta-6,22-diene-3,5,8-triol; 4: Emodine; 5: 6-hydroxyemodin (Citreorosein; 6: Chrysophanein; 7: Physcionin

A total of five pure compounds (**1** to **5**) and one mixture of two compounds (**6** and **7**) were isolated from *R. abyssinicus*. Based on their spectral data (1H and 13C NMR, 1H-1H COSY, HSQC, HMBC, and ROESY), their chemical structures as illustrated in Fig. 1 were identified as follows: **1**: Chrysophanol; **2**: Physcion; **3**: Ergosta-6,22-diene-3,5,8-triol; **4**: Emodin; **5**: 6-hydroxyemodin (Citreorosein); **6**: Chrysophanein; **7**: Physcionin. Compounds **1, 2** and **3** were derived from the EtOAc fraction while the remaining were isolated from the methanolic extract.

#### Chrysophanol (1)

Yellow powder; (C_15_H_10_O_4_); ^1^H-NMR (600 MHz, CDCl_3_) δ: 12.08 (*s*, 1-OH), 11.97 (*s*, 8-OH) 7.84 (*d*, 7.5 Hz, H-5), 7.77 (*br s*, H-6), 7.69 (*br s*, H-4), 7.30 (*d*, 8.4 Hz, H-7), 7.12 (*br s*, H-2), 2.48 (*s*, −CH_3_); ^13^C-NMR (150 MHz, CDCl_3_) δ: 192.5 (C-9), 182.1 (C-10), 162.7 (C-1), 162.4 (C-8), 149.3 (C-3), 137.0 (C-6), 133.6 (C-11), 133.2 (C-14), 124.5 (C-7), 124.3 (C-2), 121.3 (C-4), 119.9 (C-5), 115.8 (C-12), 113.7 (C-13), 22.4 (−CH_3_).

#### Physcion (2)

Yellow powder; (C_16_H_12_O_5_); ^1^H-NMR (600 MHz, CDCl_3_) δ: 12.34 (*s*, 1-OH), 12.15 (*s*, 8-OH), 7.65 (*br s*, H-5), 7.39 (*d*, 2.5 Hz, H-4), 7.10 (*br s*, H-7), 6.70 (*d*, 2.5 Hz, H-2), 3.95 (*s*, OCH_3_), 2.46 (*s*, −CH_3_); ^13^C-NMR (150 MHz, CDCl_3_) δ: 190.8 (C-9), 182.1 (C-10), 166.6 (C-3), 165.2 (C-1), 162.5 (C-8), 148.5 (C-6), 135.2 (C-14), 133.2 (C-11), 124.6 (C-7), 121.4 (C-5), 113.7 (C-12), 110.3 (C-13), 108.3 (C-4), 106.8 (C-2), 56.1(−OCH_3_), 22.2 (−CH_3_).

#### Ergosta-6,22-diene-3,5,8-triol (3)

White powder; (C_28_H_46_O_3_); ^13^C-NMR (150 MHz, CDCl_3_): δ 135.5 (C-6), 135.3 (C-22), 132.4 (C-23), 130.9 (C-7), 82.3 (C-5), 79.6 (C-8), 66.6 (C-3), 56.3 (C-17), 51.8 (C-14), 51.2 (C-9), 44.7 (C-13), 42.9 (C-24), 39.9 (C-20), 39.4 (C-11), 37.1 (C-10), 37.0 (C-4), 34.8 (C-1), 33.2 (C-25), 30.2 (C-2), 28.8 (C-15) 23.5 (C-12), 21.0 (C-21), 20.8 (C-16), 20.1 (C-26), 19.8 (C-28), 18.3 (C-19), 17.7 (C-27), 13.0 (C-18).

#### Emodin (4)

Red powder; (C_15_H_10_O_5_); ^1^H-NMR (600 MHz, DMSO-*d*_*6*_) δ: 12.1 (*s*, 3-OH), 12.0 (*s*, 8-OH) 7.48 (*d*, 0.7 Hz, H-5), 7.16 (*d*, 0.7 Hz, H-7) 7.11 (*d*, 2.4 Hz, H-4) 6.59 (*d*, 2.4 Hz, H-2), 2.41 (*s*, −CH_3_); ^13^C-NMR (150 MHz, DMSO-*d*_*6*_) δ: 190.2 (C-9), 181.9 (C-10), 166.1 (C-1), 164.9 (C-3), 161.9 (C-8), 148.7 (C-6), 135.6 (C-14), 133.3 (C-11), 124.6 (C-7), 120.9 (C-5), 113.8 (C-12), 109.4 (C-13), 109.3 (C-4), 108.4 (C-2), 21.9 (−CH_3_).

#### Citreorosein (5)

Red powder; (C_15_H_10_O_6_); ^1^H-NMR (600 MHz, CD_3_OD) δ: 7.28 (*br s*, H-2), 7.75 (*br s*, H-4), 7.20 (*br s*, H-5), 6.54 (*br s*, H-7), 4.70 (*s*, −OCH_2_-); ^13^C-NMR (150 MHz, CD_3_OD) δ: 191.5 (C-9), 183.4 (C-10), 169.1 (C-8), 166.7 (C-6), 163.7 (C-1), 152.9 (C-3), 136.9 (C-11), 135.0 (C-14), 122.2 (C-2), 118.4 (C-4), 115.9 (C-13), 111.1 (C-5), 109.2 (C-7), 108.5 (C-12), 64.1 (−OCH_2_-).

#### Chrysophanein (6)

Yellow powder; (C_21_H_20_O_9_); ^1^H-NMR (600 MHz, DMSO-*d*_*6*_) δ: 13.1 (*s*, 1-OH), 7.88 (*m*, H-5), 7.86 (*m*, H-6) 7.71 (*d*; 7.9 Hz, H-7) 7.51 (*br s*, H-4) 7.21 (*br s*, H-2) 5.20–3.10 (Glu), 2.44 (*s*, 3-CH_3_); ^13^C-NMR (150 MHz, DMSO-*d*_*6*_) δ: 188.0 (C-9), 182.6 (C-10), 162.2 (C-1), 158.7 (C-8), 148.1 (C-3), 136.4 (C-6), 135.2 (C-11), 132.6 (C-14), 124.5 (C-2), 122.9 (C-7), 121.0 (C-5), 119.8 (C-4), 115.3 (C-12), 115.2 (C-13), 101.0 (C-1′), 77.8 (C-5′), 77.0 (C-3′), 73.7 (C-2′), 70.0 (C-4′), 61.1 (C-6′), 21.9 (−CH_3_).

#### Physcionin (7)

Yellow powder; (C_22_H_22_O_10_); ^1^H-NMR (600 MHz, DMSO-*d*_*6*_) δ: 12.8 (*s*, 1-OH), 7.50 (*br s*, H-4), 7.37 (*d*, 2.3 Hz, H-5) 7.19 (*d*, 2.3 Hz, H-7) 7.18 (*br s*, H-2), 5.20–3.10 (Glu), 3.97 (*s*, −OCH_3_), 2.42 (*s*, −CH_3_); ^13^C-NMR (150 MHz, DMSO-*d*_*6*_) δ 186.9 (C-9), 182.4 (C-10), 165.2 (C-6), 162.1 (C-1), 161.2 (C-8), 147.6 (C-3), 135.1 (C-11), 132.5 (C-14), 124.7 (C-2), 119.7 (C-4), 114.96 (C-13), 114.95 (C-12), 107.9 (C-7), 106.9 (C-5), 101.1 (C-1′), 77.9 (C-5′), 77.1 (C-3′), 73.8 (C-2′), 70.3 (C-4′), 61.3 (C-6′), 56.6 (−OCH_3_) 21.8 (−CH_3_).

### Antimicrobial activity

Analysis of inhibitory parameters revealed variability in antimicrobial activity within extracts and isolated compounds, and within microbial strains tested (Table 1). Thus, all the three organic extracts exhibited activity in all the microorganisms tested, with MIC and MMC values ​​varying between 32 and 256 μg/mL. The highest activity observed with the crude extract (MIC = 32 μg/mL) was found against *C. neoformans*, and particularly the EtOAc fraction against *S. flexneri, S. aureus* and *C. albicans.* This fraction was the most active with MIC values between 32 and 64 μg/mL. Although in most cases the MMC values ​​appeared to be double the MICs, the methanolic and acetate extracts were found to be fungicidal against *C. neoformans* at 32 μg/mL while at the same concentration the EtOAc fraction was bactericidal against *S. flexneri.*

**Table 1:** Antimicrobial activity (MIC and MMC in μg/mL) of extracts and isolated compounds from *R. abyssinicus* as well as reference antimicrobial drugs

Extracts/ Compounds	Inhibition parameters	*P. aeruginosa*	*S. flexneri*	*S. aureus*	*MSSA01*	*MRSA03*	*C. albicans*	*C. neoformans*
MeOH extract	MIC	64	128	64	64	64	64	32
	MMC	128	128	128	128	128	64	32
	MMC/MIC	2	1	2	2	2	1	1
EtOAc fraction	MIC	64	32	32	64	64	32	32
	MMC	128	32	64	64	64	64	32
	MMC/MIC	2	1	2	1	1	2	1
*n*-BuOH fraction	MIC	128	128	128	256	256	64	32
	MMC	256	256	128	256	256	64	64
	MMC/MIC	2	2	1	1	1	1	2
**1**	MIC	64	32	32	64	64	64	32
	MMC	128	32	64	64	128	64	32
	MMC/MIC	2	1	2	1	2	1	1
**2**	MIC	8	8	8	16	16	8	8
	MMC	16	16	8	32	32	8	8
	MMC/MIC	2	2	1	2	2	1	1
**3**	MIC	128	128	256	>256	>256	128	64
	MMC	256	>256	>256	>256	>256	>256	128
	MMC/MIC	2	/	/	/	/	/	2
**4**	MIC	16	8	8	32	32	8	8
	MMC	16	16	8	32	32	8	8
	MMC/MIC	1	2	1	1	1	1	1
**5**	MIC	32	32	32	128	128	16	16
	MMC	64	32	32	256	256	32	32
	MMC/MIC	2	1	1	2	2	2	2
**6 + 7**	MIC	16	8	8	16	16	16	8
	MMC	16	16	8	16	32	16	8
	MMC/MIC	1	2	1	1	2	1	1
Ref^a^	MIC	0.5	8	0.5	4	4	1	2
	MMC	0.5	8	0.5	4	4	1	2
	MMC/MIC	1	1	1	1	1	1	1

/: not determined; *MIC* Minimum Inhibitory Concentration; *MMC* Minimum Microbicidal Concentration; ^a^: fluconazole for yeasts and ciprofloxacin for bacteria

With regards to the isolated compounds, their MMC were either equal to or two times higher than the corresponding MIC. The most active inhibited bacterial and fungal growth at a concentration of 8 μg/mL, and in some cases their activity was comparable to that of the reference drug. At this concentration of 8 μg/ml, the compounds **2** and **4** inhibited the growth of *C. albicans* and *C. neoformans,* and also exhibited a fungicidal activity against these strains. An inhibition of growth accompanied by microbicidal activity was noted with the compound **4** as well as the mixture **6 + 7** against *C. neoformans* and *S. aureus.* However, these compounds displayed bactericidal activity against *S. flexneri* at 16 μg/mL, i.e. twice their MIC. Similar observations were noted on compound **2** against *P. aeruginosa.* All of the strains tested were less sensitive to the compound **3**.

### Combined effect of the MeOH extract/compounds and antibiotics

The results of the interaction study between the methanolic extract/compounds (**2** and **4**), and ciprofloxacin in bacteria or fluconazole in yeasts are presented in Table 2. We found that the effect of the association of the methanolic extract with this antibacterial and antifungal agents was synergistic in nature whatever the microorganism tested. Nevertheless, the association between compound **2** and ciprofloxacin exhibited an additive effect against MSSA01 and MRSA03 strains. The interaction between compound **4** and fluconazole was also shown to be additive with respect to the yeasts *C. albicans* and *C. neoformans*. An additive effect was also observed against *C. albicans* when this compound was combined with ciprofloxacin.

**Table 2:** Interactions of the methanol extract/compounds from *R. abyssinicus* and antibiotics against bacterial and yeast species

Microorganisms	MeOH extract				Compound 2				Compound 4			
	FICA	FICEx	FIC	Interpretation	FICA	FIC2	FIC	Interpretation	FICA	FIC5	FIC	Interpretation
*P. aeruginosa*	0.25	0.125	0.37	Synergistic	0.125	0.125	0.25	Synergistic	0.25	0.125	0.25	Synergistic
*S. flexneri*	0.125	0.125	0.25	Synergistic	0.031	0.25	0.281	Synergistic	0.0625	0.25	0.31	Synergistic
*S. aureus*	0.25	0.125	0.37	Synergistic	0.25	0.125	0.375	Synergistic	0.5	0.125	0.625	Additive
*MSSA01*	0.125	0.0625	0.18	Synergistic	0.0625	0.5	0.5625	Additive	0.25	0.25	0.5	Synergistic
*MRSA03*	0.125	0.125	025	Synergistic	0.0625	0.5	0.5625	Additive	0.25	0.125	0.375	Synergistic
*C. albicans*	0.125	0.0625	0.18	Synergistic	0.0625	0.0625	0.125	Synergistic	0.25	0.5	0.75	Additive
*C. neoformans*	0.0625	0.0625	0.125	Synergistic	0.0625	0.0625	0.125	Synergistic	0.25	0.5	0.75	Additive

*FICA* MIC of antibiotic tested in combination/MIC of antibiotic tested alone; *FICEx* MIC of extract tested in combination/MIC of extract tested alone; *FIC2* MIC of compound **2** tested in combination with antibiotic/ MIC of compound **2** tested alone; *FIC4* MIC of compound **4** tested in combination with antibiotic/ MIC of compound **4** tested alone; FIC: MIC of antibiotic tested in combination/MIC of antibiotic tested alone + MIC of extract/compound tested in combination/MIC of extract/compound tested alone; antibiotics: ciprofloxacin for bacteria and fluconazole for yeasts

### Mechanisms of antibacterial activity

**Fig. 2:**
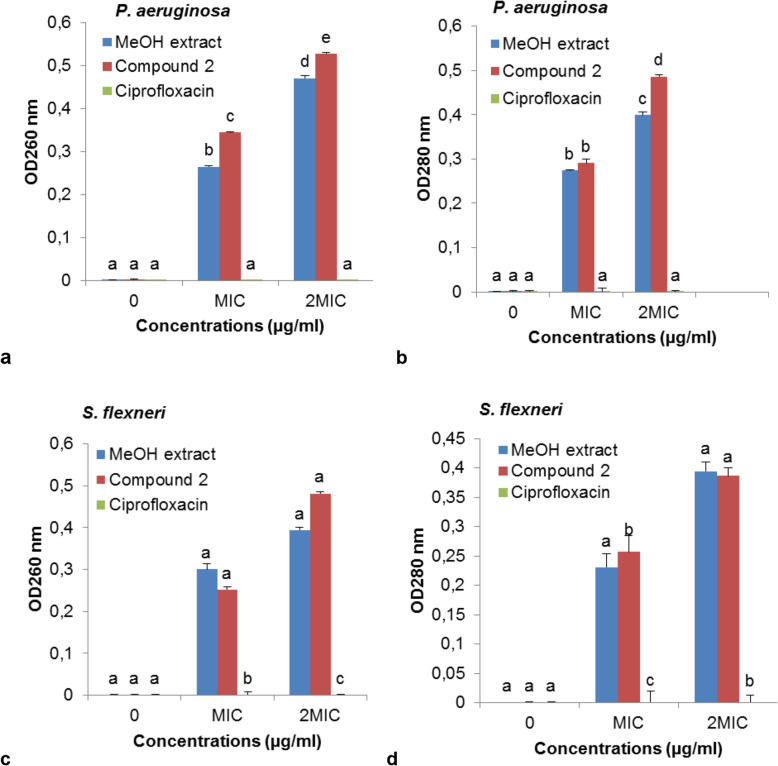
Appearance of 260 and 280 nm absorbing material in the filtrates of *P. aeruginosa* and *S. flexneri* in control suspensions and after treatment with the different concentrations of MeOH extract and compound 2

**Fig. 3:**
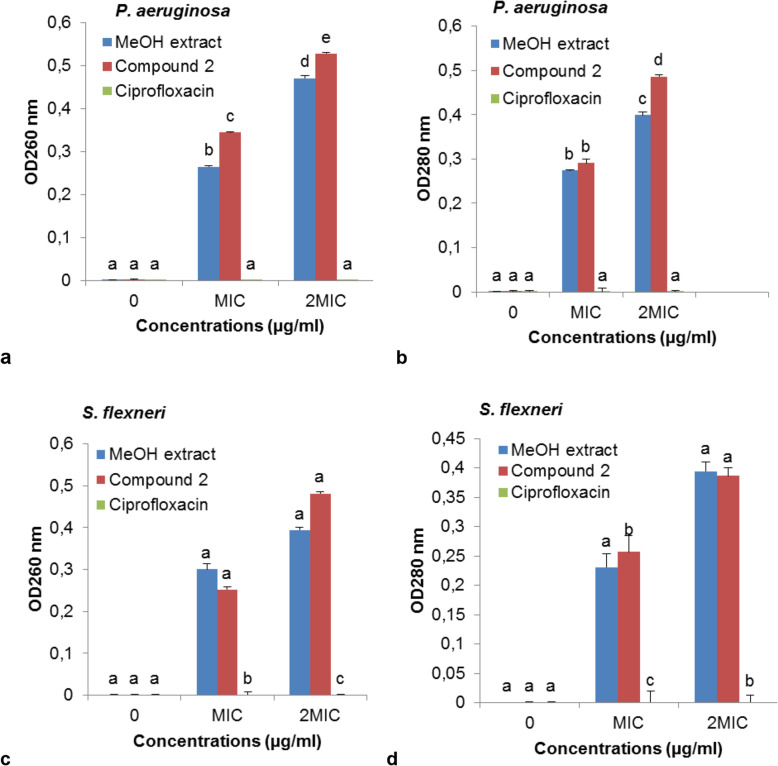
Appearance of reducing sugar (μg/mg) in the filtrates of *P. aeruginosa* (**a**) and *S. flexneri* (**b**) for control suspensions and after treatment with the different concentrations of MeOH extract and compound 2

**Fig. 4:**
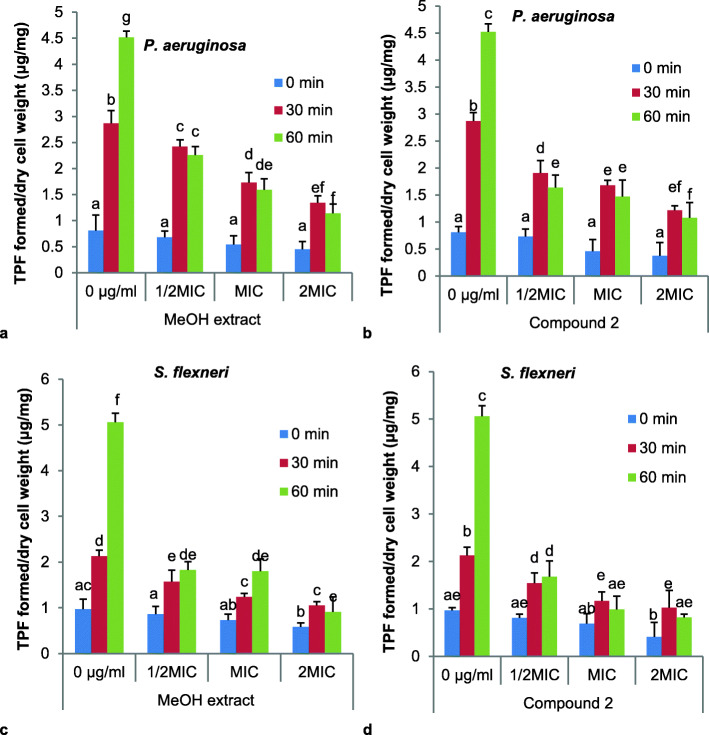
Effect of MeOH extract and compound 2 on respiratory chain dehydrogenase in *P. aeruginosa* and *S. flexneri*. TPF: TriPhenyl Formazan

The study of the mechanism of action of the methanol extract/ compound **2** in comparison with the reference antibiotic, ciprofloxacin was carried out by measuring on the one hand the optical densities at 260 and 280 nm and on the other hand the appearance of reducing sugars in the bacterial culture suspensions, and finally by measuring the activity of the respiratory chain dehydrogenase enzyme activity. These results are illustrated in Figs. 2, 3 and 4, respectively.

#### Effect on the cell membrane leakage

It was noted that the methanolic extract and the compound **2** induced leakage of biological material absorbing at 260 and 280 nm by *P. aeruginosa* (Fig. 2a and b) and *S. flexneri* (Fig. 2c and d), at concentrations equal to or two times their MIC. However, this effect of the tested samples on membrane leakage was found to be concentration dependent in the presence of both microorganisms. In the presence of *P. aeruginosa*, compound **2** displayed higher leakage effect than the crude extract.

#### Effect on the sugar leakage of membrane from the bacteria

The study of the appearance of reducing sugar also revealed that the latter increased in the bacterial culture suspensions as the concentration of the methanolic extract as well as that of the compound **2** tested increased from ½ CMI to 2 MIC through MIC (Fig. 3). The appearance of reducing sugar was also more important in the presence of the product **2** as compared to the *R. abyssinicus* extract.

#### Effect on the respiratory chain dehydrogenase enzyme activity in the bacteria

The effect of MeOH extract and compound **2** on respiratory chain dehydrogenase in *P. aeruginosa* and *S. flexneri* expressed as average TriPhenyl Formazan (TPF) formed is illustrated in Fig. 4. Generally, for a tested concentration of a compound and on a given microorganism, we observed an increase in TPF released over time. However, as the concentration of the agent tested increased, there was a significant decrease in the TPF formed for each incubation time. This reduction was more important against *S. flexneri* both for the crude extract and for the compound **2** tested.

### Antioxidant activity

Regarding the antioxidant activity, the capacity of the extract or compounds to scavenge the DPPH radical was determined and expressed in EC_50_, followed by determination of the total antioxidant capacity expressed in Gallic acid equivalent (GEAC). From these results documented in Table 3, it was observed that compound **2** displayed a significant scavenging potential against the DPPH radical (EC_50_ = 3.08 ± 0.44 μg/mL), closed to that of the reference vitamin C (1.81 ± 0.19 μg/mL). However, compounds **1** and **6 + 7** had an interesting scavenging power against the DPPH radical with EC_50_ values ​​of 4.52 ± 0.36 μg/mL and 7.63 ± 1.27 μg/mL, respectively compared to the MeOH (62.11 ± 0.39 μg/mL), EtOAc (72.29 ± 0.71 μg/mL) and *n*-BuOH (76.54 ± 0.78 μg/mL) extracts.

**Table 3:** Antioxidant activities of extracts and some isolated compounds from *R. abyssinicus*

Extracts/compounds	DPPH free radical scavenging activity (EC_50_, μg/mL)	Gallic acid equivalent antioxidant capacity (GEAC, μg/mL)
MeOH extract	62.11 ± 0.39^a^	73.23 ± 0.61^a^
EtOAc fraction	72.29 ± 0.71^b^	58.44 ± 0.38^b^
*n*-BuOH fraction	76.54 ± 0.78^c^	40.46 ± 0.74^c^
**1**	4.52 ± 0.36^d^	104.87 ± 1.43^d^
**2**	3.08 ± 0.44^e^	106.03 ± 0.87^d^
**4**	10.69 ± 0.51^f^	79.54 ± 1.26^e^
**5**	9.88 ± 0.62^f^	81.09 ± 0.93^e^
**6 + 7**	7.63 ± 1.27^g^	83.38 ± 0.22^f^
Vitamin C	1.81 ± 0.19^h^	/

EC_50_: Equivalent concentrations of test samples scavenging 50% of DPPH radical. Data represent the mean ± SD of three independent experiments carried out in triplicate. In the same column, values affected by different superscript letters (a-h) are significantly different according to one way ANOVA and Waller Duncan test; p < 0.05; /: not determined

In addition, compounds **1** and **2** displayed the highest total antioxidant capacity values of 106.03 ± 0.87 μg/mL and 104.87 ± 1.43 μg/mL, respectively. They were successively followed by the mixture of compounds **6 + 7**, compound **5**, compound **4**, then the MeOH, EtOAc and *n*-Bu-OH extracts with GEAC values ​​of 83.38 ± 0.22, 81.09 ± 0.93, 79.54 ± 1.26, 73.23 ± 0.61, 58.44 ± 0.38 and 40.46 ± 0.74 μg/mL, respectively.

### Cytotoxic activity

The cytotoxic activity of extracts and isolated compounds from *R. abyssinicus* was studied by assessing the haemolytic activity against red blood cells (RBCs) using Triton X-100 as a positive control. We observed 100% lysis with the positive control, as compared to the phosphate buffer saline (PBS) which showed no lysis of RBCs. Interestingly, none of the tested extracts and compounds showed a loss of membrane integrity as a result of cell lysis at concentrations up to 2048 μg/mL for the extracts and 256 μg/mL for the isolated compounds (results not shown).

## Discussion

The forthcome of resistant strains of bacteria and fungi against conventional antimicrobial drugs as well as side effects associated to antibiotherapy has increased the search for natural product as alternative ways to fight these organisms. Since earliest civilization, medicinal plants have been utilized by medical practitioners to treat various health related problems, and amongst these are bacterial and fungal infections [35]. Plant extracts and natural compounds are effective in the treatment of infectious diseases while at the same time alleviating many of the adverse effects associated with conventional antimicrobials [36]. This study assessed the antimicrobial and antioxidant activities of *R. abyssinicus* extracts and its isolated compounds. This plant is renowned for its multiple uses in herbal medicine to treat health issues involving oxidative stress, as well as bacterial and fungal infections.

A bio-guided fractionation of this plant was then performed to identify the potential agents endowed with antimicrobial and/or antioxidant activity. A total of five pure compounds (**1** to **5**) and one mixture of two compounds (**6** and **7**) were isolated from EtOAc fraction of *R. abyssinicus*. Compound **1,** identified as chrysophanol, was first reported from *Rheum rhabarbarum*, a herbaceous perennial plant belonging to the Polygonaceae family [37], and it has been found in various families, such as Polygonaceae, Rhamnaceae, Fabaceae, Liliaceae, Asphodelaceae, Buphorbiaceae, Meliaceae, Podocarpaceae, Picramniaceae, and Hemerocallidaceae [38, 39]. Compound **2**, physcion, is a naturally occurring anthraquinone derivative, and a major bioactive ingredient in the traditional Chinese medicine Radix and *Rhizoma rhei* [40]. It is a dihydroxyanthraquinone or 9,10-anthraquinone bearing hydroxy substituents at positions 1 and 8, a methoxy group at position 3, and a methyl group at position 6. Compound **3** was identified as ergosta-6,22-diene-3,5,8-triol, a polyhydroxysterol that has been previously isolated from *Lentinus edodes* [23]. Compound **4**, emodin, is an anthraquinone derivative that was first reported in *Aspergillus wentii*, a mycotoxin [41]. Compound **5** was identified as 6-hydroxyemodin (citreorosein), reported in the *Rumex* genus for the first time by Ertürk et al. [42]. Compound **6**, chrysophanein, is a chrysophanol glycoside that has been previously identified from leaves and roots of *Aloe hijazensis* [43]. Finally compound **7** identified as physcionin, is distributed in root of nearly all *Rheum* species [44].

All the three organic extracts exhibited activities against all the tested microorganisms, with MIC and MMC values ​​varying between 32 and 256 μg/mL. Previous studies have reported the crude extract of *R. abyssinicus* to exhibit antibacterial [15, 16], anticancer [16], antiviral [15], anti-inflammatory [15, 17], antioxidant [18], wound healing [17], antimalarial [19], diuretic and analgesic [20] activities.

However, the highest activity (MIC = 32 μg/mL) was found with the three extracts against *C. neoformans*, and particularly the EtOAc fraction against *S. flexneri, S. aureus* and *C. albicans.* This fraction was the most active with MIC values between 32 and 64 μg/mL. However, although in most cases the MMC values ​​appeared to be double the MICs, the MeOH extract and EtOAc fraction were found to be fungicidal against *C. neoformans* at 32 μg/mL while at the same concentration the ethyl acetate fraction was bactericidal against *S. flexneri.*

This study evaluated the antibacterial, antifungal and antioxidant activities of compounds isolated from *R. abyssinicus*. Most of these compounds were found to exhibit microbicidal effect with MMCs values that were either equal to or two times higher than the corresponding MICs. Compounds **2** and **4** inhibited the growth of *C. albicans* and *C. neoformans* at concentration of 8 μg/mL*,* and also exhibited a fungicidal activity against these strains. Several studies have documented a variety of pharmacological properties of physcion including laxative, hepatoprotective, antineoplastic, anti-inflammatory and anti-microbial activities [40, 45]. Compound **4**, emodin, has also been shown to display antibacterial, antifungal, antiparasitic, antioxidant, and antiviral activities [46]. The mixture of **6 + 7** also displayed considerable antimicrobial activity with bactericidal effect against *S. flexneri* at 16 μg/mL while compound **3** was inactive against most of the tested strains.

The effect of the association of the MeOH extract with ciprofloxacin and fluconazole was synergistic in nature irrespective of the microorganism tested. Compound **2** associated to ciprofloxacin exhibited synergistic effect except on MSSA01 and MRSA03 strains. A synergistic effect was also observed when compound **4** was combined with ciprofloxacin against MSSA01 and MRSA03 strains. These results indicate an increased susceptibility against the test antibiotics. Synergistic combinations have been shown to render the microorganisms extremely susceptible to concentrations of both antimicrobial agents which can be easily obtained or exceeded in the serum after administration of usual doses [47], suggesting the need of exploring the combination potential of MeOH extract, compounds **2** and **4** with reference antimicrobial drugs, to combat resistant strains. To the best of our knowledge, this is the first report of test samples and ciprofloxacin / fluconazole association against MDR strains.

This study also shows that MeOH extract and compound **2** induced leakage of biological material absorbing at 260 and 280 nm, probably nucleic acids and proteins derivatives by *P. aeruginosa* and *S. flexneri*, at concentrations equal to or two times their MIC. These observations suggest the contribution of compound **2** and MeOH extract to the alteration of microbial membrane, and the resulting leakage of intracellular material may lead to microbial death, justifying their microbicidal effect. Similar mechanisms of bacterial death have been reported in earlier studies [28]. The ability of the MeOH extract and compund **2** to alter bacterial cell membrane was further demonstrated by increased reducing sugars in the culture suspension as the concentration of both the tested MeOH extract and compound **2** raised from ½ MIC to 2 MIC through MIC. The appearance of reducing sugar was also more important in the presence of the compound **2** as compared to the *R. abyssinicus* extract. These observations have been documented with terpenoids from the leaves of *Tridax procumbens* Linn. against *E. coli* [48]. In this study, the MeOH extract of *R. abyssinicus* and compound **2** were also found to inhibit respiratory chain enzyme dehydrogenase of *P. aeruginosa* and *S. flexneri*. The decrease in the activity of this enzyme, may contribute to the inhibition of microbial growth and probably lead to death. Candidly, the inhibition of dehydrogenase activity in pathogenic bacteria is indicative of a strong antimicrobial activity since inhibition of oxido-reductases such as dehydrognases, affects respiration of the microbe.

The study of the antioxydant properties of *R. abyssinicus* extract and isolated compounds revealed the potential of the extract/isolated compounds to scavenge the DPPH radical, with compound **2** identified with highest scavenging power against the DPPH radical. Compounds **1**, **4**, **5** and **6 + 7** also displayed interesting scavenging power against the DPPH radical compared to the MeOH, EtOAc and *n*-BuOH extracts. Overall, compounds **1** and **2** had the highest total antioxidant capacity whereas the EtOAc and *n*-BuOH extracts were the least active. These observations suggest that fractionation enhanced the antioxidant activities of compounds **1**, **2**, **4**, **5** and **6 + 7** and diluted those of the EtOAc and *n*-BuOH extracts. Therefore, the presence of such compounds could be partially responsible for the antioxidant activity (AOA) found in these plant extracts; the AOA depends on the method used, reinforcing the concept that this plant extracts contain several antioxidant compounds that act in different manners. The antimicrobial and antioxidant activities of compounds **1**, **2** and **4** are in accordance with the previous studies [19, 29, 40, 46, 49]. However, this is the first report on the antibacterial, antifungal and antioxidant activities of compounds **3**, **5**–**7** against free radicals and pathogenic bacteria and fungi. The antioxidant activities of these compounds coupled to their antimicrobial properties, may offer a therapeutic option for the treatment of infectious diseases while simultaneously mitigating many of the side effects that are often associated with conventional antimicrobials [36].

## Conclusions

*R. abyssinicus* is a potential source of antibacterial, antifungal and antioxidant agents. Their mechanism of antibacterial activity is due to disruption of the cytoplasmic membrane and inhibition of the microbial respiratory chain dehydrogenase enzyme activity. Interestingly, none of the tested extracts/compounds showed cytotoxic activity against normal cells; highlighting their suitability and selectivity toward pathogenic bacteria and yeasts. The MeOH extract and compounds **2** and **4** displayed synergistic effect with the ciprofloxacin and fluconazole. The observed activity of the isolated compounds against bacteria and fungi including MDR strains deserves further exploration.

## Supplementary Information

**Additional file 1 Scheme 1** Protocol for extraction and purification of the EtOAc fraction of *Rumex abyssinicus.*

**Additional file 2 Figure S1:** ^1^H-NMR Spectrum of compound 1. **Figure S2:** ^13^C-NMR Spectrum of compound 1. **Figure S3:** HSQC Spectrum of compound 1. **Figure S4:** ^1^H-^1^HCOSY Spectrum of compound 1. **Figure S5:** HMBC Spectrum of compound 1. **Figure S6:** ^1^H-NMR Spectrum of compound 2. **Figure S7:** ^13^C-NMR Spectrum of compound 2. **Figure S8:** HSQC Spectrum of compound 2. **Figure S9:** COSY Spectrum of compound 2. **Figure S10:** HMBC Spectrum of compound 2. **Figure S11:** ^1^H-NMR Spectrum of compound 3. **Figure S12:** ^13^C-NMR Spectrum of compound 3. **Figure S13:** HSQC Spectrum of compound 3. **Figure S14:** ^1^H-^1^H COSY Spectrum of compound 3. **Figure S15:** HMBC Spectrum of compound 3. **Figure S16:** ^1^H-NMR Spectrum of compound 4. **Figure S17:** ^13^C-NMR Spectrum of compound 4. **Figure S18:** HSQC Spectrum of compound 4. **Figure S19:** ^1^H-^1^HCOSY Spectrum of compound 4. **Figure S20:** HMBC Spectrum of compound 4. **Figure S21:** ^1^H-NMR Spectrum of compound 5. **Figure S22:** ^13^C-NMR Spectrum of compound 5. **Figure S33:** HSQC Spectrum of compound 5. **Figure S24:** ^1^H-^1^H COSY Spectrum of compound 5. **Figure S25:** HMBC Spectrum of compound 5. **Figure S26:** ^1^H-NMR Spectrum of compounds 6 and 7. **Figure S27:** ^13^C-NMR Spectrum of compounds 6 and 7. **Figure S28:** HSQC Spectrum of compounds 6 and 7. **Figure S29:** ^1^H-^1^HCOSY Spectrum of compounds 6 and 7. **Figure S30:** HMBC Spectrum of compounds 6 and 7.

## Data Availability

The datasets used and analyzed during the current study are available from the corresponding author.

## Abbreviations

^13^C-NMR: Carbon Thirteen Nuclear Magnetic Resonance
^1^H-NMR: Proton Nuclear Magnetic Resonance
2D NMR: Two-dimension Nuclear Magnetic Resonance
ATCC: American Type Culture Collection
CC: Column Chromatography
COSY: Correlation Spectroscopy
DMSO: Dimethylsulfoxide
EtOAc: Ethyl acetate
HMBC: Heteronuclear Multiple Bond Connectivities
HNC: Herbier National du Cameroun
HR-EI-MS: High Resolution Electron Impact Mass Spectrometry
HR-TOFESIMS: High-Resolution Time of Flight Electrospray Ionization Mass Spectrometry
HSQC: The Heteronuclear Single Quantum Coherence
IR: Infra-red
MDR: Multi-Drug-Resistant
MeOH: Methanol
MHA: Mueller Hinton agar
MHB: Mueller Hinton broth
MIC: Minimum inhibitory concentration
MMC: Minimum Microbicidal Concentration
NA: Nutrient agar
*n*-BuOH: n-Butanol
NMR: Nuclear Magnetic Resonance
Rf: Retention factor
TLC: Thin Layer Chromatography
TMS: Tetramethylsilane
TOF-ESIMS: Time of Flight Electrospray Ionization Mass Spectrometry
UV: Ultra-violet
