# Supplementary material for: Antibacterial, antifungal and antioxidant activities of whole plant chemical constituents of Rumex abyssinicus
Source: BMC Complement Med Ther. 2021 Jun 5;21:164. doi: 10.1186/s12906-021-03325-y (PMC8180025; doi:10.1186/s12906-021-03325-y)
Supplement: Supplementary file 1 — Additional file 1 Scheme 1 Protocol for extraction and purification of the EtOAc fraction of Rumex abyssinicus. [file 12906_2021_3325_MOESM1_ESM.docx]

Extraction with MeOH (3 × 20 L, 72 h)

The whole plant of *Rumex abyssinicus* (4.5 kg)

EtOAc fraction (50 g)

C_2_

(300 mg)

Silica gel column chromatography

*n*-Hex/EtOAc95:5

Differential solubilization (of 195g) in EtOAc and *n*-BuOH

**Scheme 1** Protocol for extraction and purification of the EtOAc fraction of *Rumex abyssinicus*

Recrystallization in EtOAC

**6 + 7**

10 mg

**5**

11 mg

D_3_

(400 mg)

D_2_

(1.85 g)

D_1_

(1.40 g)

**4**

40 mg

**3**

15 mg

Sephadex LH-20 gel column chromatography in Methanol

Silica gel column chromatography

*n*-Hex/EtOAc(90:10 to 80:20)

C_1_

(500 mg)

Sephadex LH-20 gel column chromatography in Methanol

**2**

17 mg

**1**

15 mg

B (5.00 g)

F (2.05 g)

E (2.44 g)

D (1.90 g)

C (4.00 g)

G (5.21 g)

H (4.15 g)

A (4.00 g)

Fractionation using silica gel column chromatography

Residue (100.5 g)

*n*-BuOH fraction (18 g)

MeOH extract (200 g)
