# Supplementary material for: Antibacterial, antifungal and antioxidant activities of whole plant chemical constituents of Rumex abyssinicus
Source: BMC Complement Med Ther. 2021 Jun 5;21:164. doi: 10.1186/s12906-021-03325-y (PMC8180025; doi:10.1186/s12906-021-03325-y)
Supplement: Supplementary file 2 — Additional file 2 Figure S1: 1H-NMR Spectrum of compound 1. Figure S2: 13C-NMR Spectrum of compound 1. Figure S3: HSQC Spectrum of compound 1. Figure S4: 1H-1HCOSY Spectrum of compound 1. Figure S5: HMBC Spectrum of compound 1. Figure S6: 1H-NMR Spectrum of compound 2. Figure S7: 13C-NMR Spectrum of compound 2. Figure S8: HSQC Spectrum of compound 2. Figure S9: COSY Spectrum of compound 2. Figure S10: HMBC Spectrum of compound 2. Figure S11: 1H-NMR Spectrum of compound 3. Figure S12: 13C-NMR Spectrum of compound 3. Figure S13: HSQC Spectrum of compound 3. Figure S14: 1H-1H COSY Spectrum of compound 3. Figure S15: HMBC Spectrum of compound 3. Figure S16: 1H-NMR Spectrum of compound 4. Figure S17: 13C-NMR Spectrum of compound 4. Figure S18: HSQC Spectrum of compound 4. Figure S19: 1H-1HCOSY Spectrum of compound 4. Figure S20: HMBC Spectrum of compound 4. Figure S21: 1H-NMR Spectrum of compound 5. Figure S22: 13C-NMR Spectrum of compound 5. Figure S33: HSQC Spectrum of compound 5. Figure S24: 1H-1H COSY Spectrum of compound 5. Figure S25: HMBC Spectrum of compound 5. Figure S26: 1H-NMR Spectrum of compounds 6 and 7. Figure S27: 13C-NMR Spectrum of compounds 6 and 7. Figure S28: HSQC Spectrum of compounds 6 and 7. Figure S29: 1H-1HCOSY Spectrum of compounds 6 and 7. Figure S30: HMBC Spectrum of compounds 6 and 7. [file 12906_2021_3325_MOESM2_ESM.docx]

**Studies on the bioactive flavonoids isolated from *Rumex abyssinicus***

Irene Chinda Kengne^1^, Léonel Donald Tsamo Feugap^2^, Abdel Jélil Njouendou^3^, Claudia Darille Jouogo Ngnokam^2^, Mahamat Djamalladine Djamalladine^2^, David Ngnokam^2^, Laurence Voutquenne-Nazabadioko^4^ and Jean-De-Dieu Tamokou^1,^*

***Supporting information***

**List of Contents**

**Cover page**  1

**Figure S1**. ^1^H NMR Spectrum of Compound **1**  3

**Figure S2**. ^13^C NMR Spectrum of Compound **1**  3

**Figure S3**. ^1^H-^1^H COSY Spectrum of Compound **1** 4

**Figure S4**. HSQC Spectrum of Compound **1**  4

**Figure S5**. HMBC Spectrum of Compound **1** 5

**Figure S6**. ^1^H-NMR Spectrum of Compound **2** 6

**Figure S7**. ^13^C-NMR Spectrum of Compound **2** 6

**Figure S8**. ^1^H-^1^H COSY Spectrum of Compound **2** 7

**Figure S9**. HSQC Spectrum of Compound **2** 7

**Figure S10**. HMBC Spectrum of Compound **2** 8

**Figure S11**. ^1^H-NMR Spectrum of Compound **3** 9

**Figure S12**. ^13^C-NMR Spectrum of Compound **3** 9

Figure S13. ^1^H-^1^H COSY Spectrum of Compound 3 10

**Figure** **S14**. HSQC Spectrum of Compound **3** 10

**Figure S15**. HMBC Spectrum of Compound **3** 11

**Figure S16**. 1H-NMR Spectrum of Compound **4** 12

**Figure S17**. 13C-NMR Spectrum of Compound **4** 12

**Figure S18**. 1H-1H COSY Spectrum of Compound **4** 13

**Figure S19**. HSQC Spectrum of Compound **4** 13

**Figure S20**. HMBC Spectrum of Compound **4** 14

**Figure S21**. 1H-NMR Spectrum of Compound **5** 15

**Figure S22**. 13C-NMR Spectrum of Compound **5** 15

**Figure S23**. 1H-1H COSY Spectrum of Compound **5** 16

**Figure S24**. HSQC Spectrum of Compound **5** 16

**Figure S25**. HMBC Spectrum of Compound **5** 17

**Figure S26**. 1H-NMR Spectrum of Compounds **6** and **7** 18

**Figure S27**. 13C-NMR Spectrum of Compounds **6** and **7** 18

**Figure S28**. 1H-1H COSY Spectrum of Compounds **6** and **7** 19

**Figure S29**. HSQC Spectrum of Compounds **6** and **7** 19

**Figure S30**. HMBC Spectrum of Compounds **6** and **7** 20


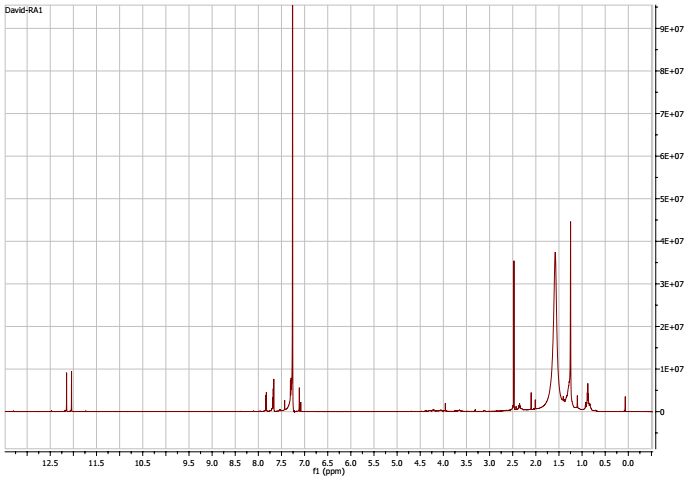


**Figure S1:** ^1^H-NMR Spectrum of compound 1


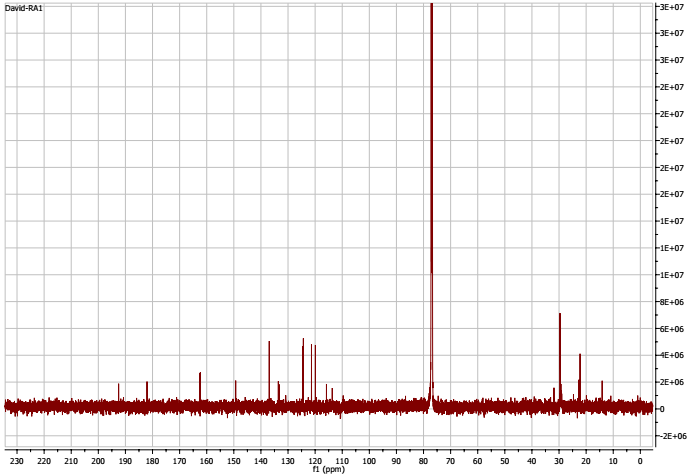


**Figure S2:** ^13^C-NMR Spectrum of compound 1


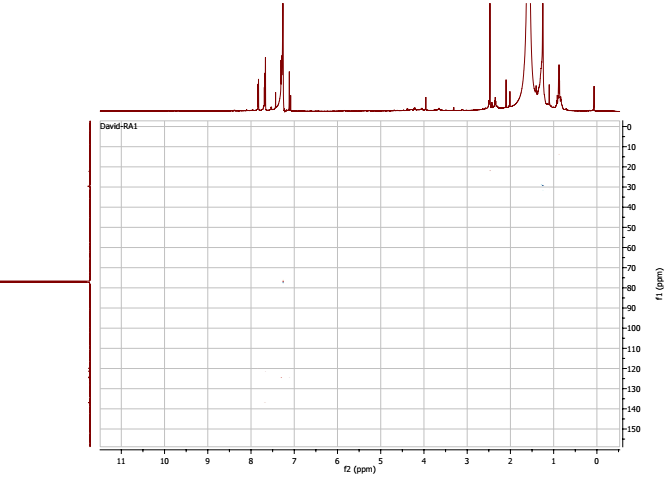


**Figure S3:** HSQC Spectrum of compound 1


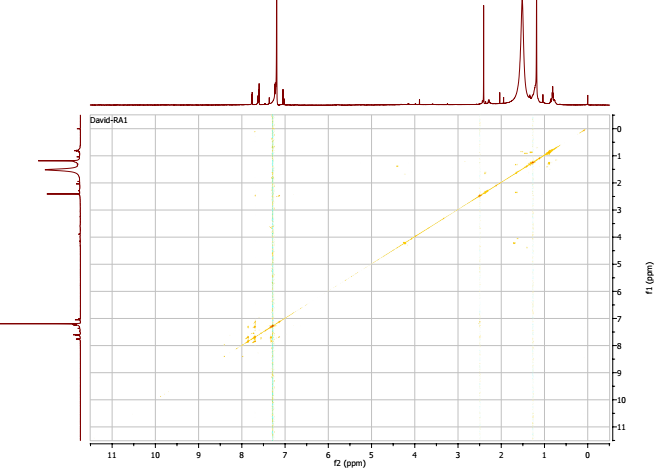


**Figure S4:** ^1^H-^1^HCOSY Spectrum of compound 1


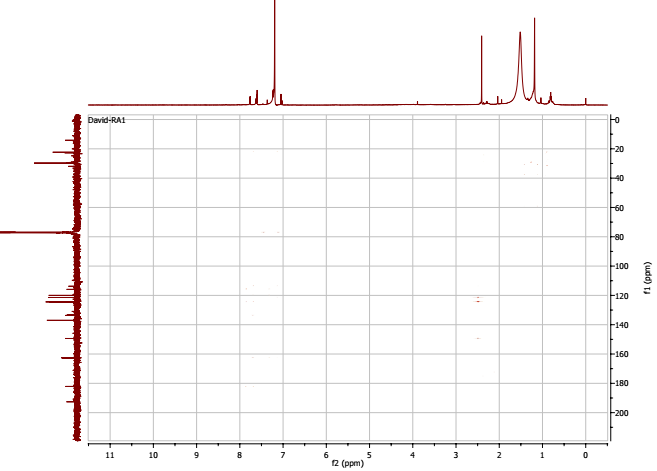


**Figure S5:** HMBC Spectrum of compound 1


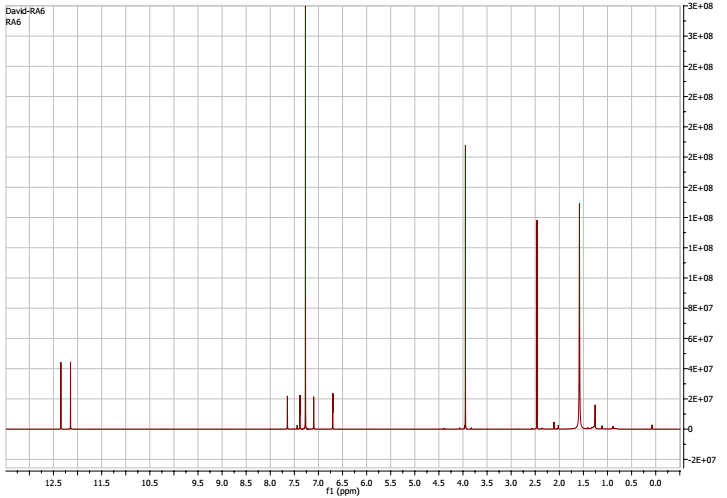


**Figure S6:** ^1^H-NMR Spectrum of compound 2


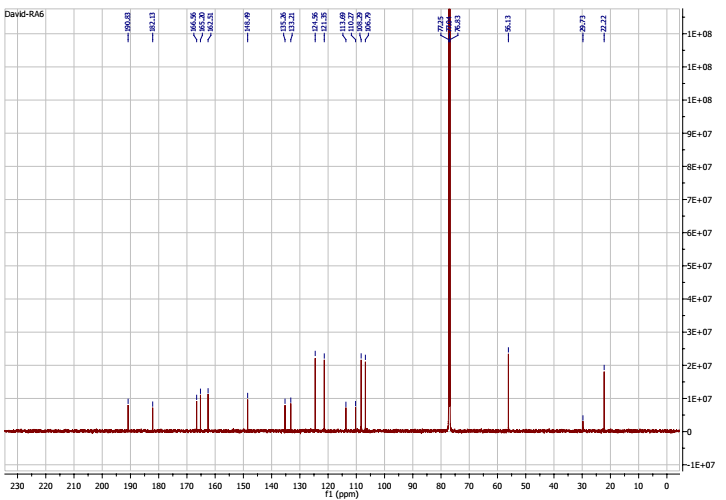


**Figure S7:** ^13^C-NMR Spectrum of compound 2


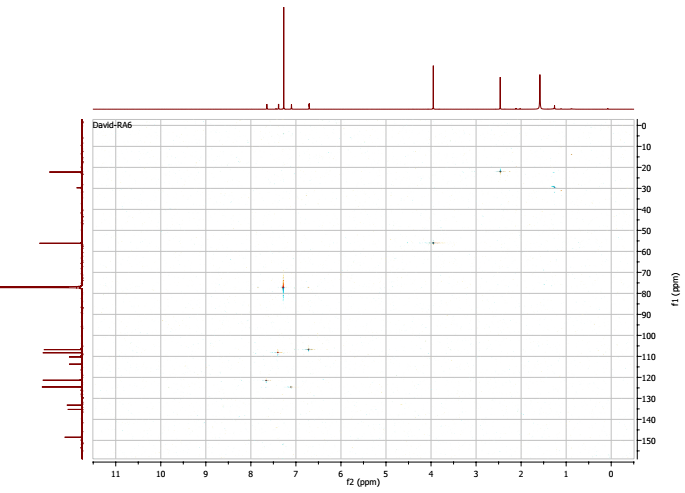


**Figure S8:** HSQC Spectrum of compound 2


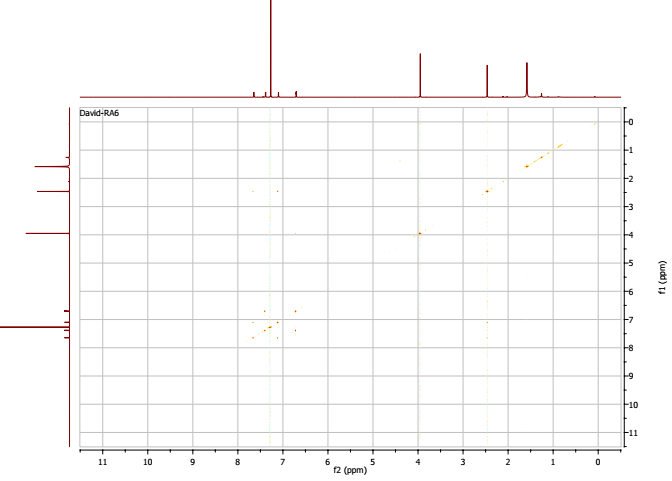


**Figure S9:** COSY Spectrum of compound 2


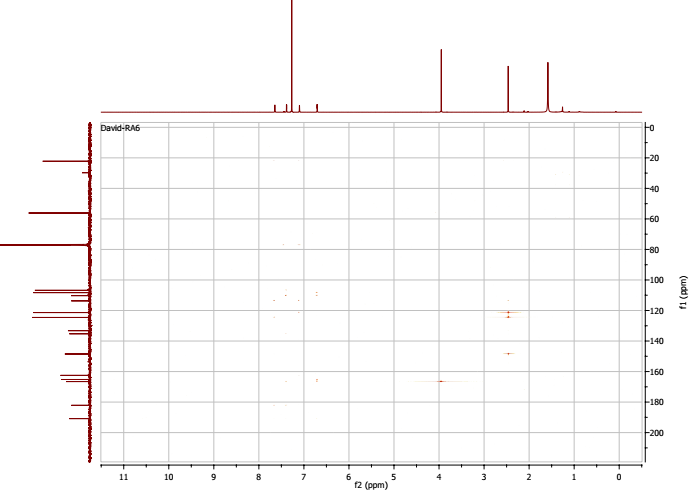


**Figure S10:** HMBC Spectrum of compound 2


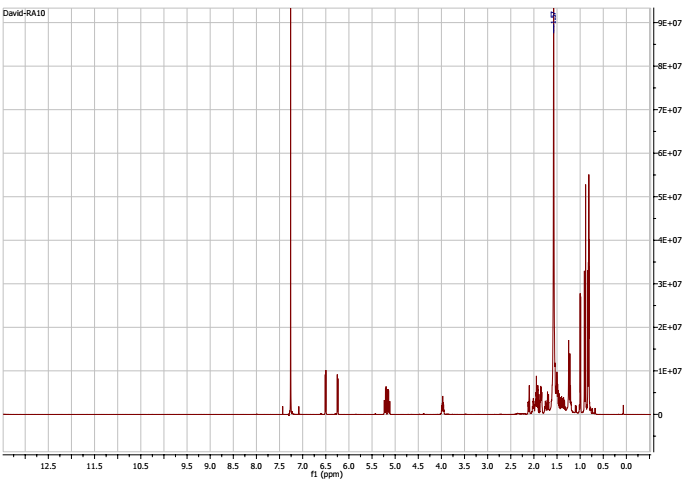


**Figure S11:** ^1^H-NMR Spectrum of compound 3


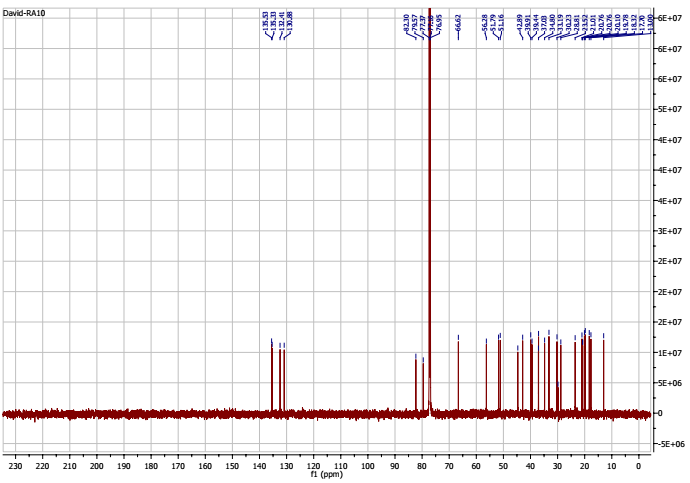


**Figure S12:** ^13^C-NMR Spectrum of compound 3


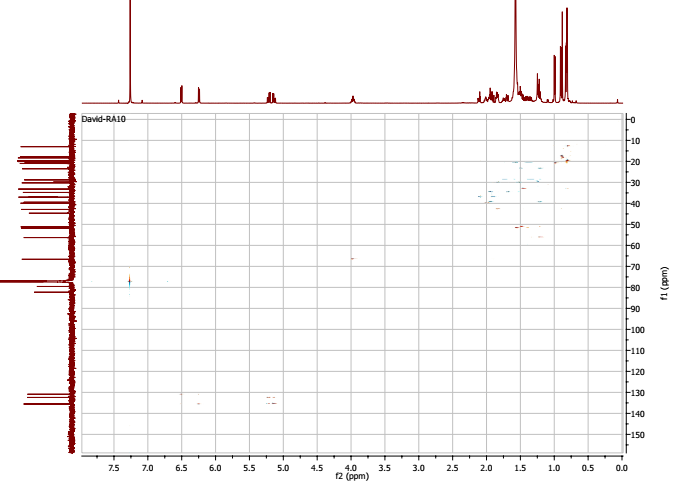


**Figure S13:** HSQC Spectrum of compound 3


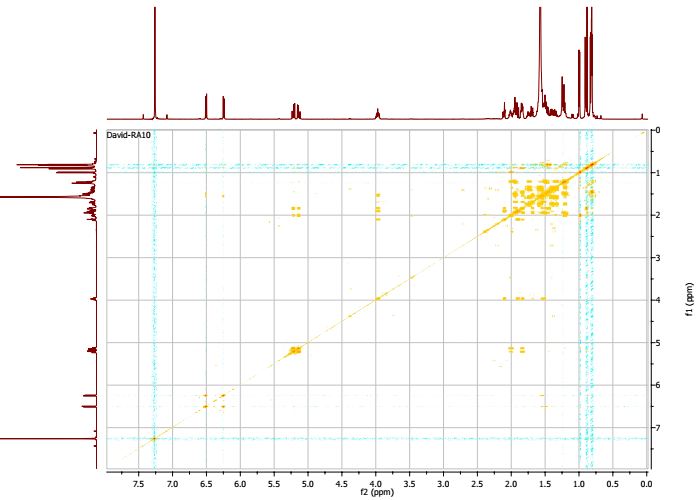


**Figure S14:** ^1^H-^1^H COSY Spectrum of compound 3


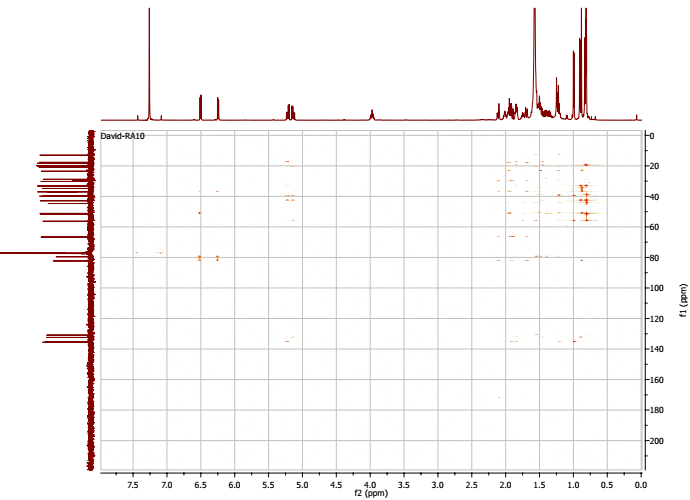


**Figure S15:** HMBC Spectrum of compound 3


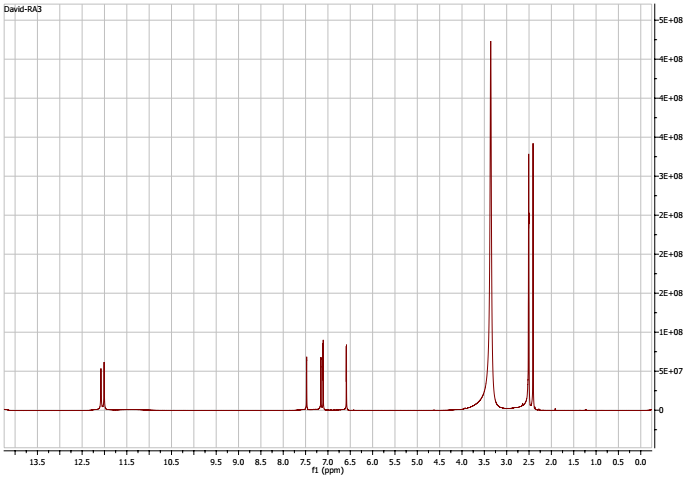


**Figure S16:** ^1^H-NMR Spectrum of compound 4


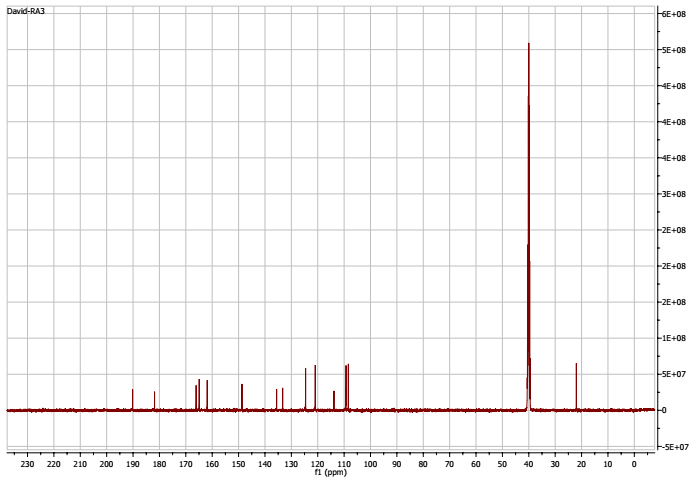


**Figure S17:** ^13^C-NMR Spectrum of compound 4


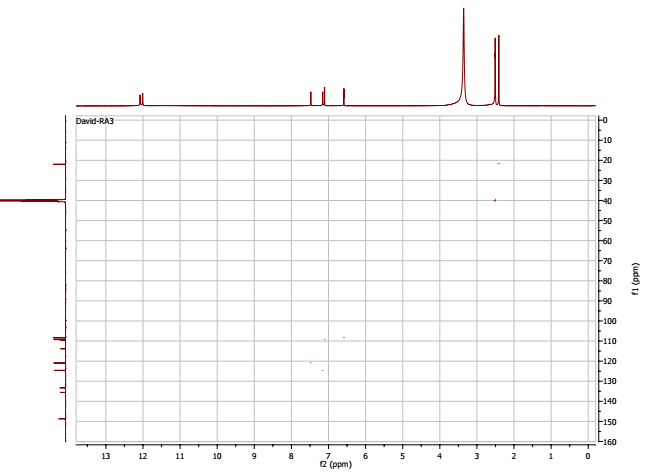


**Figure S18:** HSQC Spectrum of compound 4


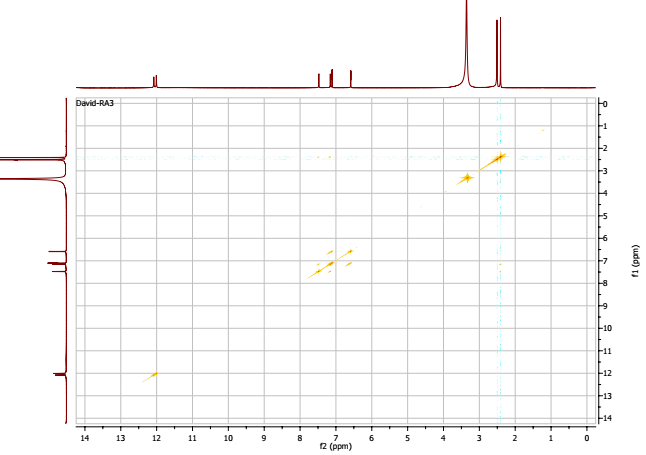


**Figure S19:** ^1^H-^1^HCOSY Spectrum of compound 4


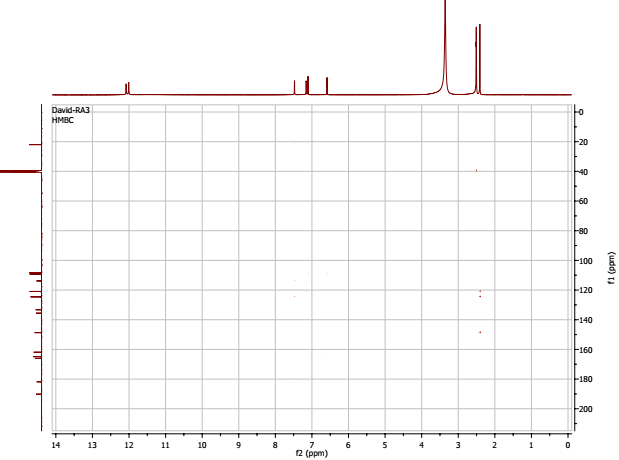


**Figure S20:** HMBC Spectrum of compound 4


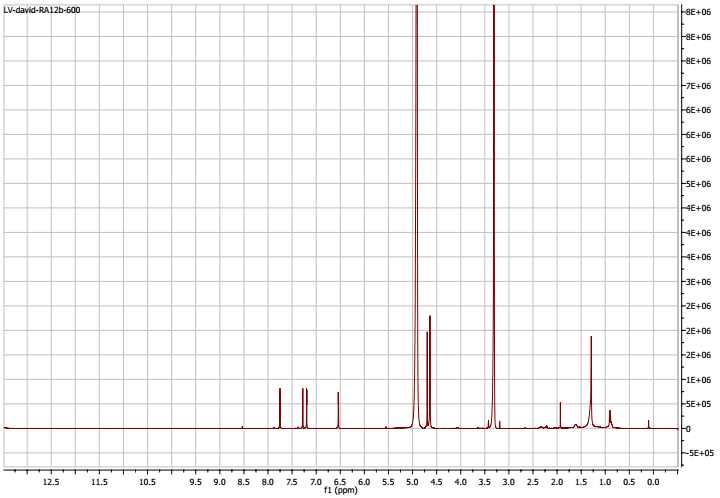


**Figure S21:** ^1^H-NMR Spectrum of compound 5


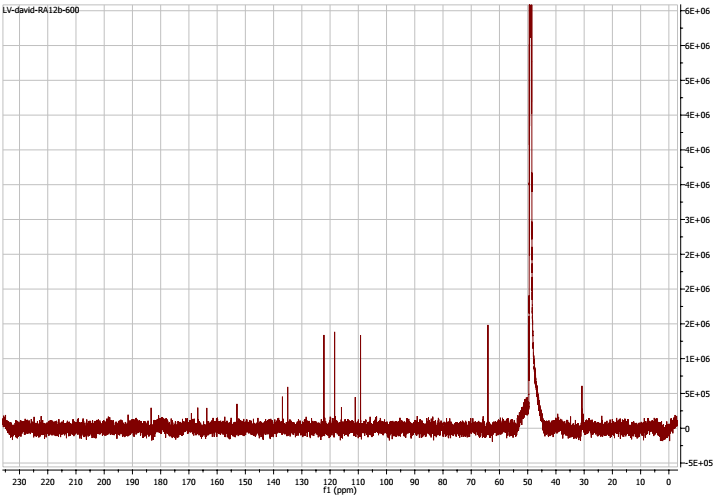


**Figure S22:** ^13^C-NMR Spectrum of compound 5


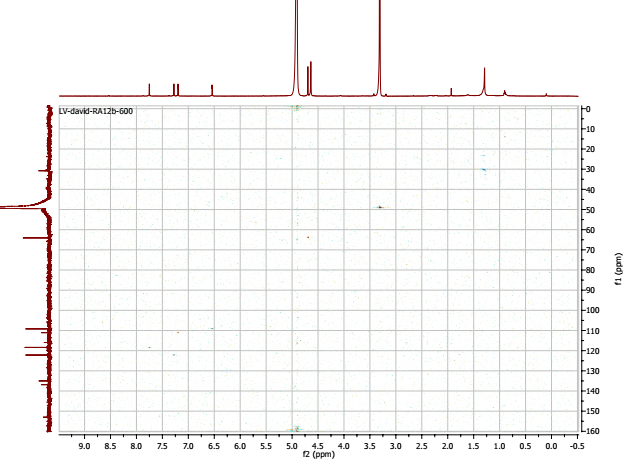


**Figure S33:** HSQC Spectrum of compound 5


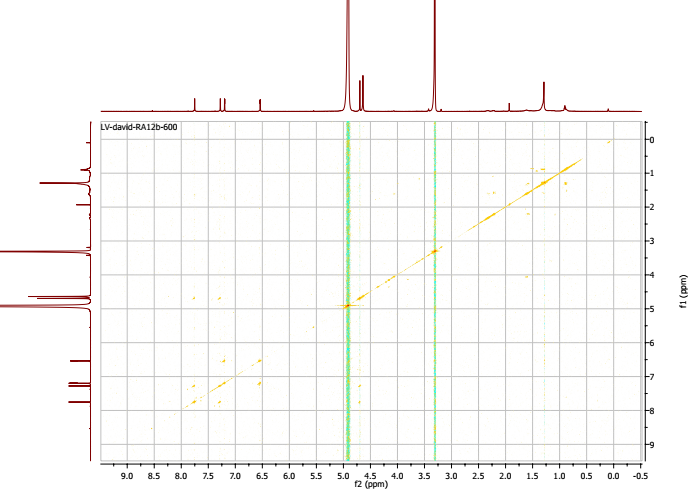


**Figure S24:** ^1^H-^1^H COSY Spectrum of compound 5


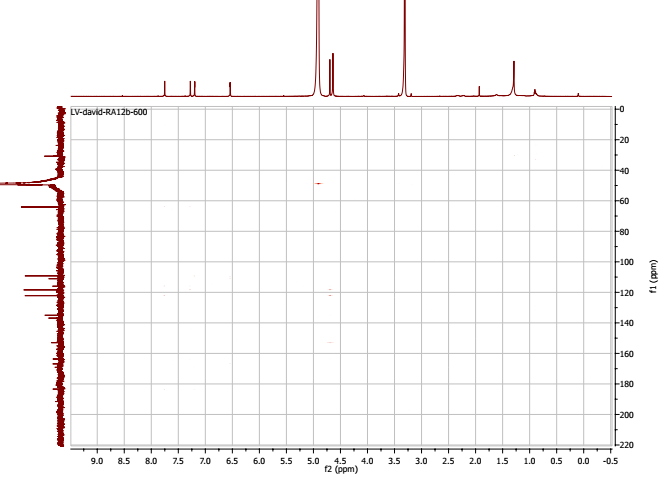


**Figure S25:** HMBC Spectrum of compound 5


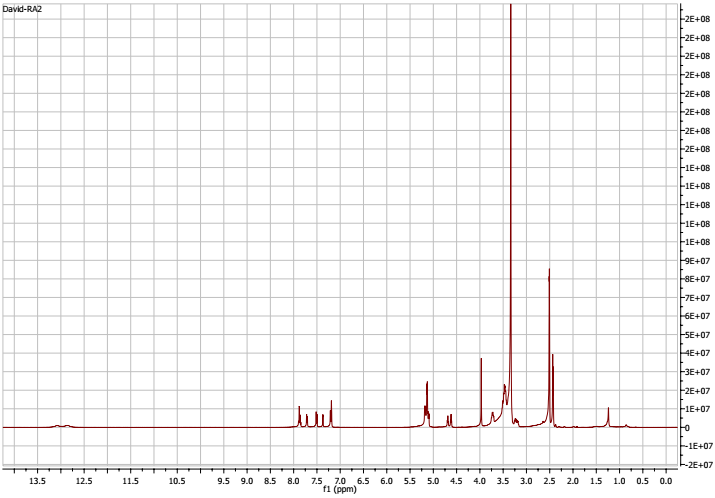


**Figure S26:** ^1^H-NMR Spectrum of compounds 6 and 7


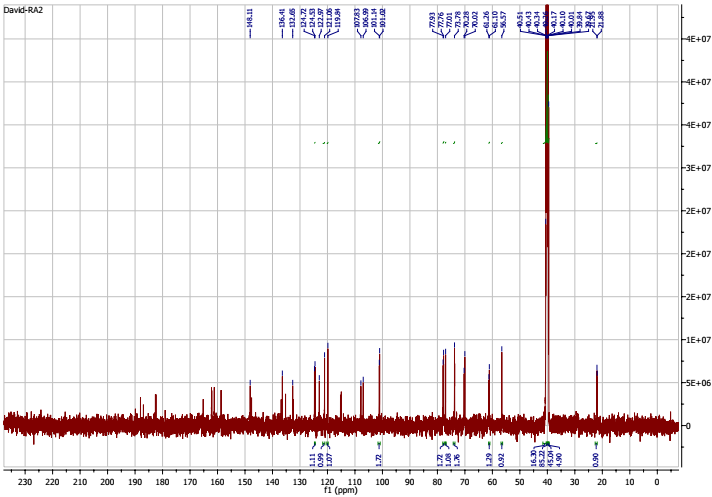


**Figure S27:** ^13^C-NMR Spectrum of compounds 6 and 7


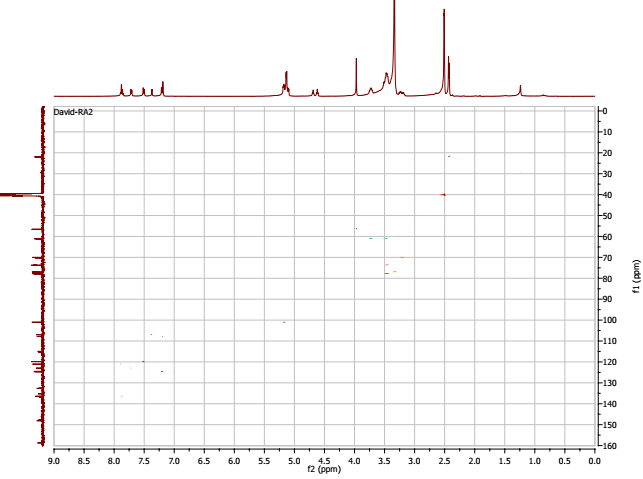


**Figure S28:** HSQC Spectrum of compounds 6 and 7


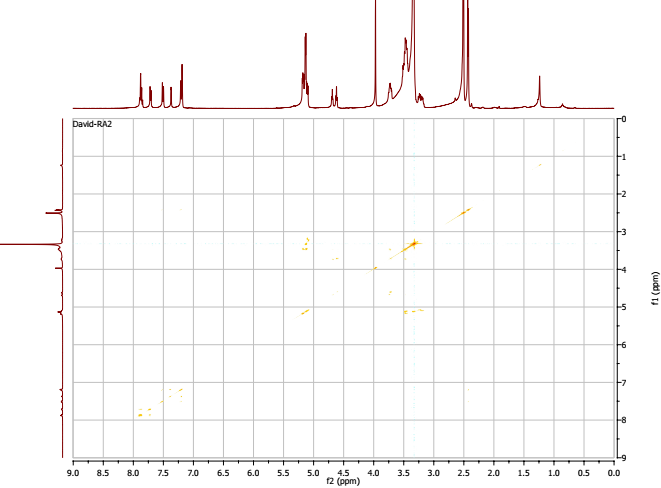


**Figure S29:** ^1^H-^1^HCOSY Spectrum of compounds 6 and 7


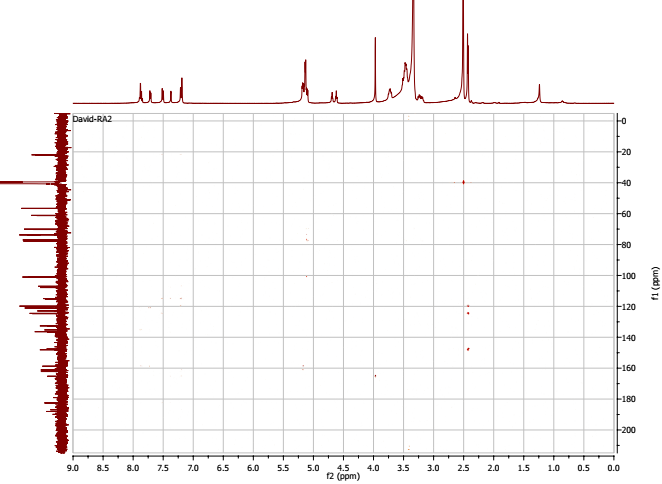


**Figure S30:** HMBC Spectrum of compounds 6 and 7
